# Drug Properties Prediction Based on Deep Learning

**DOI:** 10.3390/pharmaceutics14020467

**Published:** 2022-02-21

**Authors:** Soyoung Yoo, Junghyun Kim, Guang J. Choi

**Affiliations:** 1 Department of Bigdata Engineering, Soonchunhyang University, Asan-si 31538, Korea; yooso0731@gmail.com; 2 Department of Medical Sciences, Soonchunhyang University, Asan-si 31538, Korea; guangchoi@sch.ac.kr; 3 Department of Pharmaceutical Engineering, Soonchunhyang University, Asan-si 31538, Korea

**Keywords:** deep learning, imbalanced data, small data, principal component analysis, Wasserstein GAN, pharmaceutical formulation

## Abstract

In recent research on the formulation prediction of oral dissolving drugs, deep learning models with significantly improved performance compared to machine learning models were proposed. However, the performance degradation due to limitations of an imbalanced dataset with a small size and inefficient neural network structure has still not been resolved. Therefore, we propose new deep learning-based prediction models that maximize the prediction performance for disintegration time of oral fast disintegrating films (OFDF) and cumulative dissolution profiles of sustained-release matrix tablets (SRMT). In the case of OFDF, we use principal component analysis (PCA) to reduce the dimensionality of the dataset, thereby improving the prediction performance and reducing the training time. In the case of SRMT, the Wasserstein generative adversarial network (WGAN), a neural network-based generative model, is used to overcome the limitation of performance improvement due to the lack of experimental data. To the best of our knowledge, this is the first work that utilizes WGAN for pharmaceutical formulation prediction. Experimental results show that the proposed methods have superior performance than the existing methods for all the performance metrics considered.

## 1. Introduction

The pharmaceutical industry still strongly relies on traditional trial-and-error experiments, which are time-consuming, cost-inefficient, and unpredictable. Over recent years, in order to reduce the degree of trial-and-error in drug discovery and development, research results on data-based prediction technologies including machine learning and deep learning have been published [1,2,3,4,5,6]. In particular, deep learning has been successfully applied to drug discovery and repositioning, such as drug–target interaction prediction and drug screening, due to its ability to automatically extract nonlinear features from complex input data [7,8,9].

In the field of formulation prediction, recent research [10] showed that a deep neural network (DNN) outperforms an artificial neural network (ANN) with a single hidden layer for predicting disintegration time on orally disintegrating tablets. Previously, two prediction models were proposed in [11] for the formulations of oral fast disintegrating films (OFDF) and sustained release matrix tablets (SRMT), which are drugs that disintegrate in the oral cavity. Six machine learning methods, multiple linear regression (MLR), partial least squared regression (PLSR), support vector machine (SVM), ANNs, random forest (RF), and k-nearest neighbors (k-NN), were considered to compare with DNNs. The simulation results confirmed that the performance of the DNN model has the best performance in both cases [11]. In addition, the maximum dissimilarity algorithm with the small group filter and representative initial set selection (MD-FIS) algorithm tried to overcome the limitation in the pharmaceutical formulation prediction field, where only small and imbalanced datasets are provided due to limited experimental data. The MD-FIS algorithm is a modification of the maximum dissimilarity algorithm [12] and is a technique for selecting test data that adequately represents the characteristics of the entire data. The models proposed in the previous research outperform other machine learning models, and especially show high prediction accuracy on the training data, but do not guarantee sufficient performance on the test data. Moreover, they still do not fundamentally solve the limitation of a small dataset.

In this paper, we utilize principal component analysis (PCA) [13] and Wasserstein generative adversarial network (WGAN) [14] to improve the performance of existing models in [11]. PCA is a technique for maximizing variance and selecting essential features and was used in a recent study [15] to solve the multivariate pneumonia classification problem. WGAN is a technology that generates new samples from an original dataset. Recently, WGAN was used to improve the predictive performance of cancer diagnosis by solving the data imbalance problem [16]. In our work, for OFDF, we remove the correlation between features of the data using PCA and then predict the disintegration time using a new neural network model with a simpler and more effective structure than the existing model. For SRMT, we increase the size of the training data with WGAN and then predict the cumulative dissolution profiles for four time points (2, 4, 6, and 8 h) using the proposed neural network. The simulation results show that the two proposed models significantly improved the performance of the existing models in [11].

## 2. Proposed Models

**Figure 1:**
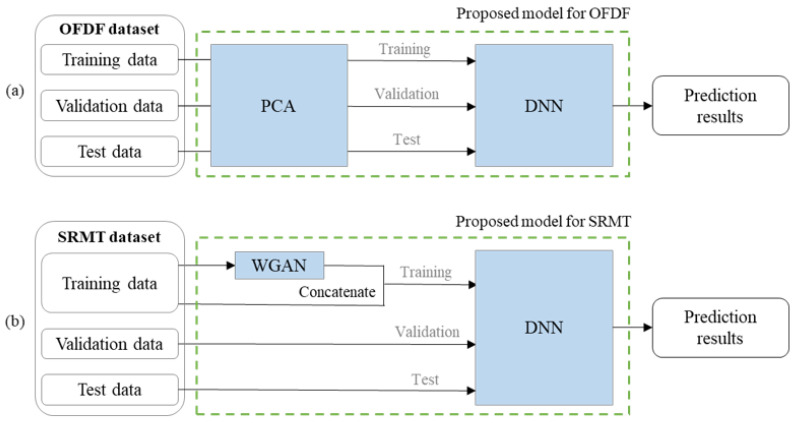
Architecture of two proposed models. (**a**) OFDF; (**b**) SRMT.

In this section, we propose two prediction models which have better performance than the existing models in [11] for OFDF and SRMT. Figure 1 represents the architectures of the proposed models. The proposed model for OFDF improves the prediction accuracy by reducing the dimensionality of the features using PCA. The proposed model for SRMT improves the prediction accuracy by increasing the number of data samples through WGAN, a data augmentation scheme. In addition, we redesign the neural network structures for both prediction models, which not only guarantee good performance but also significantly reduce the training time.

### 2.1. A Proposed Model for OFDF

The model for OFDF implements a single output regression analysis method that predicts the disintegration time using 24 feature values. Since deep learning methods generally include nonlinear modules and perform feature extraction automatically, they are relatively less susceptible to multicollinearity that causes performance degradation in linear regression models. However, if highly correlated features are used as input to the neural network, the problem of convergence to local minima can frequently occur during the training process. Therefore, we utilize PCA to transform the input data into a new representation of independent variables in the data preprocessing. The PCA is a dimensionality reduction technique that extracts only meaningful components of the entire data through orthogonal transformations. Using this technique, we can reduce the size of the original dataset as well as remove highly correlated variables.

The model for OFDF includes a neural network structure with three hidden layers and one output layer. The neural network has 50, 25, 16, and 1 neurons in that order. In the first hidden layer, dropout is performed at a rate of 0.05. The dropout is a technique for temporarily ignoring neurons in a neural network during the training. Using this technique, we prevent overfitting on the training data and improve the performance of the test data. As activation functions, the ReLU function [17] is used for all the hidden layers, and sigmoid function [18] is used for the output layer. Biases of all the layers are initialized to zeros, and weights have random values following *He* uniform distribution [19] for the initialization. The ReLU function is widely used in deep neural networks due to its effect of addressing the vanishing gradient problems. The *He* uniform is an initialization function proposed to improve the convergence speed of deep neural networks using the RELU function.

### 2.2. A Proposed Model for SRMT

The model for SRMT implements a multi-output regression analysis method that predicts the cumulative dissolution profiles for each time point (2, 4, 6, and 8 h) with 21 feature values. It requires a relatively large amount of data compared to the model for OFDF because there are 4 target variables to be predicted, but the original training data consists of only 105 data samples. We note that the training data of OFDF consists of 91 data samples. Therefore, we generated new data samples using WGAN (Wasserstein generative adversarial network), one of the data augmentation techniques, for model training. WGAN is an advanced model of vanilla generative adversarial network (GAN) [20] in which two neural networks, a generator, and a discriminator, are trained in an adversarial relationship. WGAN uses a new cost function using Wasserstein distance to address the vanishing gradient problem and mode collapse problem.

In our model, both the generator and critic of WGAN are implemented as DNNs. The generator has 6 hidden layers with 500, 250, 200, 150, 100, and 50 nodes, respectively, and the batch normalization process is performed for each hidden layer. It generates 25-dimensional data from input data of 100 dimensions randomly generated from a normal distribution. The critic has 4 hidden layers with 150, 120, 100, and 50 nodes, respectively. In the first and second hidden layers, dropout is performed at a rate of 0.1.

The new data generated by the WGAN model is combined with the original training data and used for the training of the new prediction model. The prediction model consists of five hidden layers and one output layer. Each contains 150, 130, 100, 50, 30, and 4 neurons. Biases of all the layers are initialized to zeros, and weights have random values following a random normal distribution as initial values. As activation functions, the ReLU function is used for all the hidden layers, and the sigmoid function is used for the output layer. In the first and second hidden layers, dropout is performed at a rate of 0.1.

## 3. Experiment and Performance Evaluation

In this section, we present datasets used in the experiment and evaluation criteria for two prediction models. We compare the performance of the proposed models and the existing models in [11] through experimental results. For a fair comparison, we used the same datasets in [21] and evaluation criteria considered in [11] for all experiments.

### 3.1. Dataset

Two datasets used in the experiment contain 131 formulations for OFDF and 145 formulations for SRMT. OFDF has disintegration time and SRMT has cumulative dissolution profiles (2, 4, 6, and 8 h) as target variables to be predicted. For representing the properties of active pharmaceutical ingredients (APIs), molecular descriptors were used. All formulations were described with the nine molecular descriptors including molecular weight, $X\log P_{3}$, hydrogen bond donor count, hydrogen bond acceptor count, rotatable bond count, topological polar surface area, heavy atom count, complexity, and logS. The excipient types were encoded to different numbers. The process parameters were also considered. The parameters for OFDF are weight, thickness, tensile strength, elongation, folding endurance, and actual drug content, and the parameters for SRMT are granulation process, diameter, and hardness. Therefore, OFDF consists of 24 feature variables and 1 target variable, and SRMT consists of 21 feature variables and 4 target variables.

The authors in [11] performed the MD-FIS algorithm twice to extract 20 data samples each for the validation and test sets that adequately characterize the overall data, and classified the rest as the training data. As a result, the training, validation, and test datasets of OFDF were divided into 91, 20, and 20 data samples, and those of SRMT were divided into 105, 20, and 20 samples.

The training data is used for model training, the validation data is used for tuning for model optimization, and the test data is used to check the performance of a new dataset that is not used for the training process. For effective model training, we normalized feature values and target values. For instance, the target value of the OFDF has a real value from 0 to 1, indicating 0 to 100 s. The cumulative dissolution profile value of SRMT has a value from 0 to 1, representing 0% to 100%.

### 3.2. Evaluation Criteria

Instead of the commonly used correlation and determinants as performance metrics for evaluating regression problems in machine learning, we use the following specific criteria introduced in the previous research [11].

In the pharmaceutical field, the case where the absolute error is less than 10% is generally assumed to be a successful prediction [10], so we used 10 s, which is about 10% of the maximum observation, as the criterion for the OFDF prediction model. The accuracy is expressed as the ratio of the case where the difference between the predicted value and the actual value is within 10 s among all predictions. The definition is expressed in Equation (1):

(1) $${\mathrm{Accuracy}}_{\mathrm{OFDF}}=\frac{\mathrm{Number}(|f{}^{\prime }-f|\leq 10)}{\mathrm{All}\;\mathrm{predictions}},$$

where $f^{\prime }$ is a predicted value and *f* is an actual experimental value.

In the prediction model for SRMT, the performance is evaluated using the similarity factor $f_{2}$ [22] to measure the similarity according to the recommendations of the FDA (US Food and Drug Administration). When the absolute error of dissolution profile prediction is 10%, the corresponding $f_{2}$ value is about 50, and the value increases as the error decreases. Therefore, a case where the $f_{2}$ value is greater than or equal to 50 is considered a successful prediction. The accuracy of the prediction is expressed as the ratio of successful predictions among all predictions. This definition is expressed in Equation (2):

(2) $${\mathrm{Accuracy}}_{\mathrm{SRMT}}=\frac{\mathrm{Number}(f_{2}\geq 50)}{\mathrm{All}\;\mathrm{predictions}}.$$

### 3.3. Performance Evaluation for OFDF

We utilized PCA to reduce the training time and improve the performance of the prediction model for OFDF. The PCA reduces the dimensionality by projecting a high-dimensional space into a low-dimensional space so that the low-dimensional representation retains some meaningful properties of the original data, thereby eliminating unnecessary dimensions and reducing the training time for the model. Therefore, it is possible to achieve high performance at a faster speed than when using raw data. In addition, the multicollinearity problem can be alleviated by removing highly correlated variables.

The number of principal components can be selected using a scree plot or cumulative percentage of variance (CPV). In order to select an appropriate number of dimensions before reducing the dimension of data using PCA, we drew a scree plot with the number of components on the *X*-axis and eigenvalues on the *Y*-axis.

**Figure 2:**
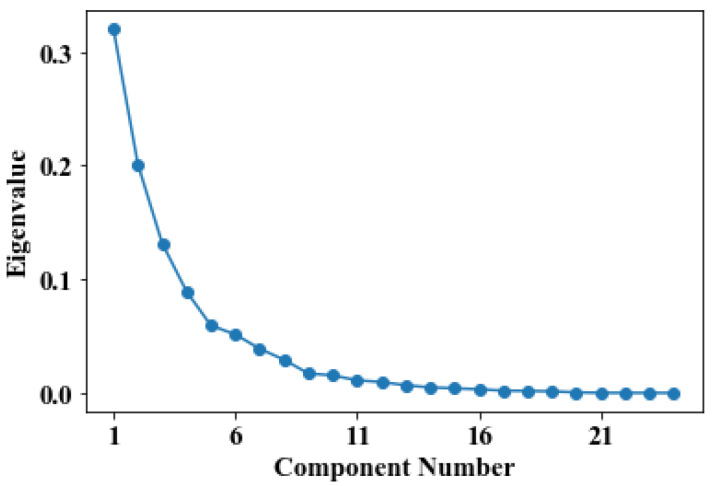
Scree plot of OFDF dataset.

Figure 2 is a scree plot of the OFDF dataset. In the figure, the elbow point is observed as 5, which indicates that the 21-dimensional data can be reduced to 5-dimensional data through PCA. Here, the elbow point represents the point at which the slope softens after a certain point on the scree plot. We used five principal components in order from the first principal component as input data of the proposed model. Specifically, we found an appropriate principal component using only the training data and applied the same setting to the validation data and test data.

**Figure 3:**
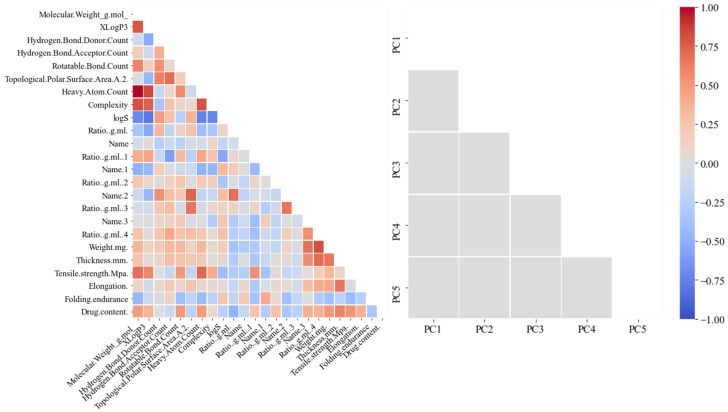
Correlation matrices of OFDF datasets before and after applying PCA.

Figure 3 shows correlation coefficients between variables in the training data before and after applying PCA. As the color of a square gets closer to red or blue from gray, it is a variable pair that has a strong correlation. Although there were several variable pairs with strong correlation in the training data before applying PCA, it is confirmed that multicollinearity between variables is removed through PCA.

In order to verify the effect of PCA on the performance improvement, we trained the proposed DNN model using each of the training data before and after applying PCA. For the training, mean square error (MSE) was used as the loss function and Adam [23] was used as the optimization algorithm. The learning rate was set to 0.01. The number of epochs was set to 800 and the batch size was set to 32. Here, an epoch is a unit in which one training is performed on the entire training dataset, and a batch is the bundle of training data samples used to update the parameters.

Table 1, Table 2 and Table 3 show the numbers of trainable parameters for the existing OFDF model, the proposed OFDF model without PCA, and the proposed OFDF model with PCA, respectively. The number of trainable parameters means the number of parameters that are iteratively updated during the model training. Table 4 compares the total number of trainable parameters, the total number of updates, and the training time of the three models above. The training time refers to the time it takes to find an optimal or near-optimal value after inputting the training data into each deep learning model. We note that, as the number of trainable parameters or the total number of updates increases, the complexity of the model increases, which leads to an increase in the training time.

The DNN model proposed in this paper has a shallow but effective structure, so the number of trainable parameters is reduced to about 10% of the existing model. In addition, by adjusting the batch size, the total number of updates is also reduced to about 3% of the existing model. This shortens the training time by reducing the computational complexity. In the case of applying PCA to the proposed model, the number of trainable parameters is reduced by 950 because the dimension of the input data is reduced from 24 to 5. As a result, the training time was significantly reduced.

Table 5 is the performance evaluation result of the three models. Compared with the existing OFDF models, the two proposed OFDF models show better performance for all the training, validation, and test data. In particular, the proposed OFDF model with PCA has the best accuracy with a 20% increase compared to the existing OFDF model in the test data. Moreover, in terms of the training time, the proposed OFDF model with PCA was the best among the three models. Therefore, we propose a final model of the entire structure in which the given data is preprocessed with PCA and then the newly designed neural network is trained using it.

### 3.4. Performance Evaluation for SRMT

We addressed several data augmentation techniques to improve the performance of a prediction model for SRMT. A commonly used data augmentation technique is an oversampling technique. However, since the dataset for SRMT is non-categorical data, it is difficult to directly apply the oversampling. Therefore, we multiplied the final cumulative dissolution rates by 10 and removed the decimal portion to generate labels for the oversampling. These labels are represented by 10 classes with integer values between 0 and 9. We applied oversampling techniques, random over-sampling (ROS), synthetic minority over-sampling technique (SMOTE) [24], and adaptive synthetic sampling (ADASYN) [25], to the categorically re-expressed dataset. As a result, the training data increased from 105 samples to 200, 200, and 203 samples, in that order. Table 6 shows the number of data per class in the training data. Through the oversampling techniques, it can be seen that data samples of each class are generated in the same or similar number as the seventh class, which has the largest number of samples.

**Figure 4:**
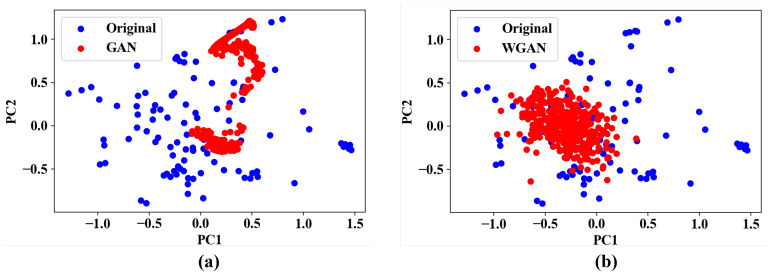
Distribution of data samples generated by (**a**) GAN and (**b**) WGAN.

As another data augmentation technique, we considered GAN and its variant, WGAN. WGAN uses a critic instead of a discriminator of GAN and a new cost function using Wasserstein distance instead of binary cross-entropy of GAN. First, we trained GAN 5000 times to generate 2000 data samples. However, some of them did not satisfy the cumulative characteristics. Table 7 shows two cases of whether the cumulative characteristic is satisfied or not. For example, in the first sample in the Dissatisfaction column in Table 7, the cumulative dissolution amount increased to 0.798 after 6 h, and then decreased to 0.718 after 8 h. However, it is practically not possible. Therefore, we removed 309 samples that do not satisfy the cumulative characteristic among the newly generated 2000 samples. Next, in order to confirm the distribution of the generated data samples, each sample was expressed as coordinates in two-dimensional space with two principal components as each axis through PCA. Figure 4a shows the distribution of data samples generated by GAN. From the figure, it can be observed that the generated data samples are concentrated in a specific part rather than in a form that is widely spread in two-dimensional space. To solve this mode collapse problem, we generated new data samples using WGAN. As with GAN, only 351 samples that satisfy the cumulative characteristic among the generated data samples were selected. Figure 4b shows the distribution of data samples generated by WGAN. Unlike when using GAN, the distribution of the generated data samples is wide and evenly spread in two-dimensional space. From the result, it can be seen that WGAN solved the mode collapse problem.

The proposed model for SRMT is optimized with the Adam algorithm with a learning rate of 0.001 and trained 2000 times by using MSE as the cost function. In order to obtain the best performance, we set the batch size to 32.

Table 8 and Table 9 show the numbers of trainable parameters for the existing SRMT model and the proposed SRMT model, respectively. Table 10 compares the total number of trainable parameters, the total number of updates, and the training time of two models. The total number of trainable parameters of the proposed model is about five times that of the existing model, but the total number of updates is significantly reduced. As a result, the training time was reduced to less than half. This means that our proposed SRMT model converges to good performance with fewer updates even though the number of trainable parameters is greater than that of the existing model.

We also compared the performance after training the model using the datasets generated by ROS, SMOTE, ADASYN, and WGAN. Table 11 shows the experimental results for the existing SRMT model and the proposed SRMT model with data augmentation techniques. The performance of the proposed model is better than that of the existing model in most cases, and especially when using WGAN, it shows the best performance with a 10% increase compared to the existing SRMT model in the test data. Therefore, we propose a final model that augments the given training data utilizing WGAN and then uses it to train a newly designed neural network.

## 4. Conclusions

In this paper, we dealt with how to effectively utilize imbalanced datasets with small sizes for predicting the properties of drugs. For OFDF, we used PCA to remove correlation between variables and designed an effective neural network structure. For SRMT, which requires more complex and accurate predictive capability than OFDF, we used WGAN to augment data and designed an efficient neural network structure. In both cases, it was confirmed through experiments that the proposed models showed superior performance to the existing models. We note that, in the SRMT case, there is still room for further improvement since the difference in prediction accuracy between the training data and the test data is about 30%. We expect that the proposed models and techniques will contribute to facilitating data-based decision-making, speeding up the process, and reducing the rate of failure in all stages of drug discovery and development.

## Figures and Tables

**Table 1:** The number of trainable parameters of the existing OFDF model [11].

Layer	Shape	Trainable Parameters
Input	24	0
Hidden layer 1	50	1250
Hidden layer 2	50	2550
Hidden layer 3	50	2550
Hidden layer 4	50	2550
Hidden layer 5	50	2550
Hidden layer 6	50	2550
Hidden layer 7	50	2550
Hidden layer 8	50	2550
Hidden layer 9	50	2550
Hidden layer 10	50	2550
Output	1	51
**Total**		**24,251**

**Table 2:** The number of trainable parameters of the proposed OFDF model without PCA.

Layer	Shape	Trainable Parameters
Input	24	0
Hidden layer 1	50	1250
Hidden layer 2	25	1275
Hidden layer 3	16	416
Output	1	17
**Total**		**2958**

**Table 3:** The number of trainable parameters of the proposed OFDF model with PCA.

Layer	Shape	Trainable Parameters
Input	24	0
Hidden layer 1	50	300
Hidden layer 2	25	1275
Hidden layer 3	16	416
Output	1	17
**Total**		**2008**

**Table 4:** A comparison of the total number of trainable parameters, total number of updates, and training time between the existing OFDF model [11], the proposed OFDF model without PCA, and the proposed OFDF model with PCA.

Model		Total Number of Trainable Parameters	Total Number of Updates	Training Time (min:s)
Ref. [11]		24,251	81,900	01:47
Proposed OFDF Model	Without PCA	2958	2400	00:05
	With PCA	2008	2400	00:04

**Table 5:** Performance comparison between the existing OFDF model [11], the proposed OFDF model without PCA, and the proposed OFDF model with PCA.

Model		Training Data			Validation Data			Test Data		
		Accuracy (%)	RMSE	MAE	Accuracy (%)	RMSE	MAE	Accuracy (%)	RMSE	MAE
Ref. [11]		97.8	0.0382	0.0277	80	0.0765	0.0629	75	0.1009	0.0720
Proposed OFDF model	Without PCA	100	0.0256	0.0192	85	0.0755	0.0598	85	0.0813	0.0536
	With PCA	100	0.0338	0.0243	95	0.0568	0.0427	95	0.0843	0.0578

**Table 6:** The number of samples for each class of the original training data for SRMT and the new training data with the oversampling techniques.

Class	Original Training Data	ROS	SMOTE	ADASYN
1	5	20	20	21
2	4	20	20	19
3	7	20	20	21
4	8	20	20	21
5	9	20	20	21
6	13	20	20	22
7	20	20	20	20
8	15	20	20	21
9	9	20	20	18
10	15	20	20	19
**Total**	**105**	**200**	**200**	**203**

**Table 7:** Two cases of whether cumulative characteristic is satisfied in the cumulative dissolution profile for SRMT.

Satisfaction				Dissatisfaction			
**2 h**	**4 h**	**6 h**	**8 h**	**2 h**	**4 h**	**6 h**	**8 h**
0.070	0.234	0.824	0.855	0.244	0.513	**0.798**	**0.718**
0.302	0.575	0.785	0.910	0.365	0.316	**0.580**	**0.575**
0.100	0.386	0.934	0.942	0.358	0.469	**0.728**	**0.696**
0.214	0.305	0.409	0.441	**0.368**	**0.271**	**0.572**	**0.571**
0.131	0.328	0.455	0.497	**0.453**	**0.248**	**0.688**	**0.652**

**Table 8:** The number of trainable parameters of the existing SRMT model [11].

Layer	Shape	Trainable Parameters
Input	21	0
Hidden layer 1	30	660
Hidden layer 2	30	930
Hidden layer 3	30	930
Hidden layer 4	30	930
Hidden layer 5	30	930
Hidden layer 6	30	930
Hidden layer 7	30	930
Hidden layer 8	30	930
Hidden layer 9	30	930
Output	4	124
**Total**		**8224**

**Table 9:** The number of trainable parameters of the proposed SRMT model [11].

Layer	Shape	Trainable Parameters
Input	21	0
Hidden layer 1	150	3300
Hidden layer 2	130	19,630
Hidden layer 3	100	13,100
Hidden layer 4	50	5050
Hidden layer 5	30	1530
Output	4	124
**Total**		**42,734**

**Table 10:** A comparison of the total number of trainable parameters, total number of updates, and training time between the existing SRMT model [11], the proposed SRMT model.

Model	Total Number of Trainable Parameters	Total Number of Updates	Training Time (min:s)
Ref. [11]	8224	273,000	02:47
Proposed SRMT Model	42,734	37,500	01:04

**Table 11:** Performance comparison between the existing SRMT model [11] and the proposed SRMT model with data augmentation techniques.

Model		Training Data			Validation Data			Test Data		
		Accuracy (%)	RMSE	MAE	Accuracy (%)	RMSE	MAE	Accuracy (%)	RMSE	MAE
Ref. [11]		99.05	0.0384	0.0283	65	0.1625	0.1053	60	0.2019	0.1264
Proposed SRMT model	ROS	99	0.0176	0.0079	65	0.1347	0.9090	65	0.1116	0.0833
	SMOTE	100	0.0082	0.0045	65	0.1469	0.0990	55	0.1282	0.0993
	ADASYN	100	0.0073	0.0038	65	0.1438	0.0965	60	0.1292	0.0992
	WGAN	100	0.0134	0.0101	75	0.1142	0.0762	70	0.1086	0.0831

## Data Availability

The datasets used in this study are available at https://github.com/yylonly/DeepPharm-InVitro (accessed on 3 February 2022).
